# Npl3 stabilizes R‐loops at telomeres to prevent accelerated replicative senescence

**DOI:** 10.15252/embr.201949087

**Published:** 2020-02-06

**Authors:** Lara Pérez‐Martínez, Merve Öztürk, Falk Butter, Brian Luke

**Affiliations:** ^1^ Institute of Molecular Biology (IMB) Mainz Germany; ^2^ Institute of Developmental Biology and Neurobiology (IDN) Johannes Gutenberg Universität Mainz Germany

**Keywords:** Npl3, R‐loop, RNA–DNA hybrid, senescence, telomere, Chromatin, Epigenetics, Genomics & Functional Genomics

## Abstract

Telomere shortening rates must be regulated to prevent premature replicative senescence. TERRA R‐loops become stabilized at critically short telomeres to promote their elongation through homology‐directed repair (HDR), thereby counteracting senescence onset. Using a non‐bias proteomic approach to detect telomere binding factors, we identified Npl3, an RNA‐binding protein previously implicated in multiple RNA biogenesis processes. Using chromatin immunoprecipitation and RNA immunoprecipitation, we demonstrate that Npl3 interacts with TERRA and telomeres. Furthermore, we show that Npl3 associates with telomeres in an R‐loop‐dependent manner, as changes in R‐loop levels, for example, at short telomeres, modulate the recruitment of Npl3 to chromosome ends. Through a series of genetic and biochemical approaches, we reveal that Npl3 binds to TERRA and stabilizes R‐loops at short telomeres, which in turn promotes HDR and prevents premature replicative senescence onset. This may have implications for diseases associated with excessive telomere shortening.

## Graphical abstract

Npl3 interacts with telomeres through TERRA and RNA‐DNA hybrids. By stabilizing RNA‐DNA hybrids at short telomeres Npl3 drives telomere recombination and prevents accelerated senescence.

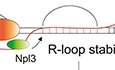

## Introduction

Telomeres, the terminal structures of linear chromosomes, promote genome integrity by preventing unscheduled DNA damage responses at chromosome ends 1, 2. Telomeres are subject to the end replication problem, which results in progressive telomere attrition with each cell division 3. In cancer cells, germ cells, and stem cells, telomerase can maintain telomere length 4, but the majority of human cells do not harbor sufficient activity to prevent telomere shortening 5. When telomeres reach a critically short length, an irreversible checkpoint‐mediated cell cycle arrest ensues 6, 7, 8. This phenomenon, called replicative senescence (senescence induced through progressive telomere shortening), serves as a tumor suppressor mechanism by limiting the proliferative potential of a transformed cell 9. On the other hand, the accumulation of senescent cells can contribute to organismal aging 10. Therefore, the rate of telomere shortening must be balanced to prevent premature senescence and aging, which may occur when critically short telomeres arise too early, while at the same time limit cell division potential to prevent cancer development.

In telomerase‐negative budding yeast, one critically short telomere is sufficient to trigger replicative senescence 11 while approximately five short telomeres are required in human cells 12. It is therefore essential to repair short telomeres that may spontaneously arise in early population doublings after telomerase loss to prevent premature senescence. In yeast, it has been demonstrated that homology‐directed repair (HDR) can prevent the early onset of senescence 13 by promoting telomere recombination at critically short telomeres 14. In addition, it has been recently shown that telomere repeat‐containing RNA (TERRA) also contributes to the regulation of HDR at critically short telomeres 15. TERRA is a long non‐coding RNA transcribed at all telomeres by RNA polymerase II 16, 17, 18. TERRA has a tendency to form R‐loops 19, 20, 21, likely due to the thermal stability of G‐rich RNAs engaged in hybrids. Telomeric R‐loops promote HDR both in pre‐senescent cells, to prevent premature replicative senescence, as well as in post‐senescent cells where telomeres are exclusively maintained through HDR 20, 22. In pre‐senescent yeast cells, R‐loops specifically accumulate at critically short telomeres and assist in defining which telomere requires HDR‐mediated elongation 15. R‐loop accumulation at shortened telomeres occurs due to the altered regulation of TERRA hybrids at short, compared to long, telomeres 15. At long telomeres, RNase H2 and Rat1 are recruited to telomeres and degrade TERRA and R‐loops, respectively, throughout the S phase. The association of RNase H2 and Rat1 is defective at short telomeres, hence allowing TERRA and R‐loop accumulation 15. Other factors that may contribute to R‐loop regulation, in the context of telomeres, have remained elusive.

Here, we investigate telomere‐associated proteins using quantitative interactomics. We identified nuclear protein localization 3 (Npl3), among others, as a telomere binding protein. We show that Npl3 associates with telomeres in a TERRA‐dependent manner, which promotes its accumulation at critically short telomeres. Our data indicate that Npl3 presence at short telomeres contributes to R‐loop stability and thereby promotes telomere recombination to prevent premature senescence. These results add to our mechanistic understanding of how R‐loop regulation promotes the repair of short telomeres by HDR during senescence and may also be relevant for the repair of double‐strand breaks (DSBs), where R‐loops have recently been demonstrated to promote recombination‐based repair 23, 24, 25.

## Results and Discussion

### Identification of telomere‐associated proteins in *Saccharomyces cerevisiae*

**Figure 1:**
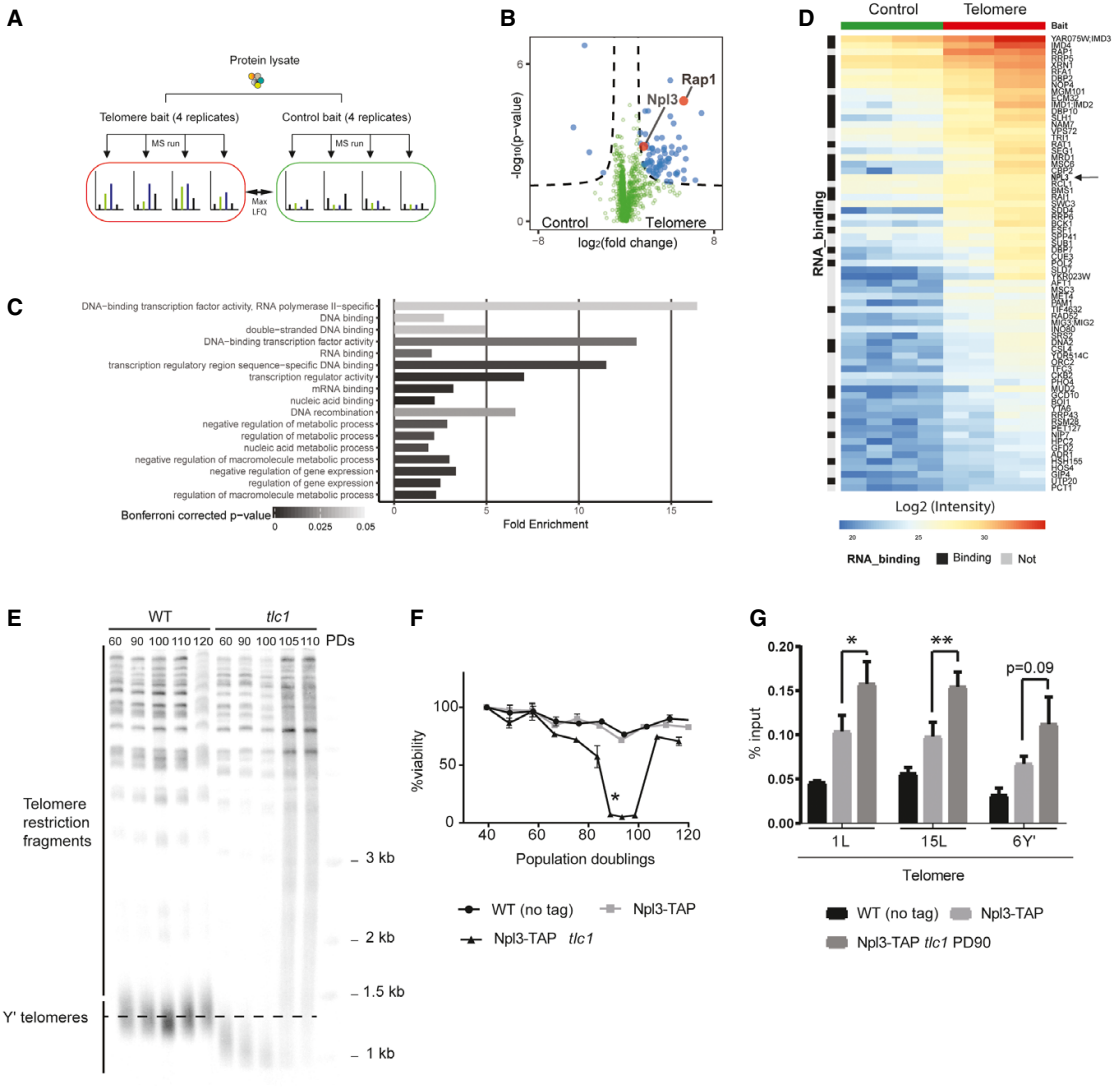
A screen for telomere‐associated proteins in yeast Schematic representation of the quantitative interactomics approach to identify proteins binding to the *Saccharomyces cerevisiae* telomeric sequence.Volcano plot of quantified proteins. Log_2_ fold change was determined as the difference between the mean LFQ intensity of the four replicates of telomeric to control sequence, and *P*‐values were calculated with a Welch *t*‐test. Proteins above the threshold *P* = 0.05, *s*0 = 1, *c* = 0.5 (dashed line) are considered enriched.Gene Ontology enrichment analysis of the telomere sequence‐associated interactors. Analysis was performed using the PantherDB.org overrepresentation test. Fisher's exact test was subjected to Bonferroni correction.Heatmap of LFQ intensity of proteins enriched at DNA baits. Telomere interactors are indicated on the right side of the heatmap. Color code indicates measured log_2_ LFQ intensities in the telomere pull‐down. RNA‐binding proteins are marked with a black box on the left side of the figure.Critically short telomeres arise after 90 population doublings in the absence of telomerase (*tlc1*). Genomic DNA was extracted at indicated population doublings and digested with XhoI. A radioactive probe was used to recognize telomeric DNA in a Southern blot.
*tlc1* cells reach senescence at PD 80–100. %viability of indicated strains after propagation in YPD is indicated at different population doublings *n* = 3. Asterisk indicates population doubling used in (G) to determine binding of Npl3 to short telomeres by TAP‐ChIP. Three independent spores from the indicated genotypes were used. Population doublings are counted from germination of meiotic products and hence begin at approx. PD40.Npl3‐TAP associates strongly to short telomeres. Cross‐linked samples from indicated strains were used in a TAP‐ChIP. Enrichment at telomeres was determined by quantitative PCR on indicated telomeres. Data represent mean % input ± SEM *n* = 3 (paired *t*‐test, one‐tailed *P < 0.05, **P < 0.01). PD90 refers to 90 population doublings in the absence of *TLC1* (telomerase RNA subunit). The no tag control serves as an indicator of non‐specific background signal.Data information: LFQ, label‐free quantification; MS, mass spectrometry; PD, population doubling; RBP, RNA‐binding protein; TAP, tandem affinity purification tag.

**Figure EV1:**
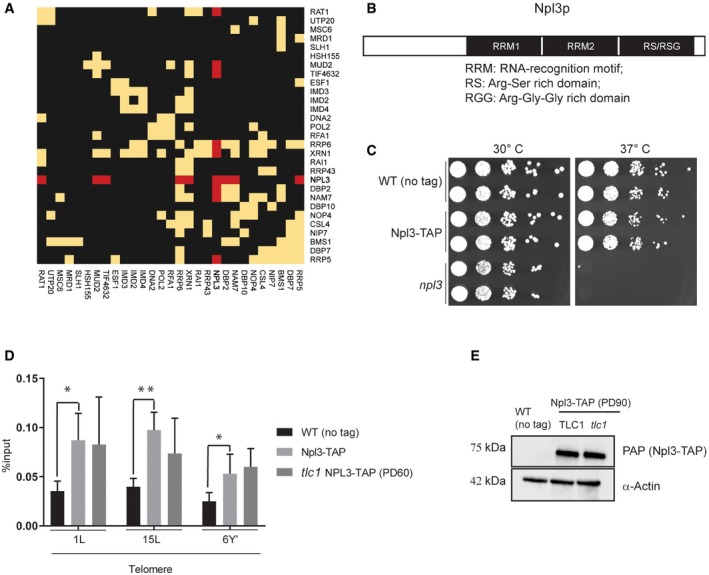
A screen for telomere‐associated proteins in yeast RBP telomere interactors from WT cell lysates form interaction clusters. Biogrid protein interactions are represented as a Heatmap. Yellow is used for presence, and black is used for absence of an annotated interaction. Clustering is performed using the complete data based on binary distance. Npl3 interactors are highlighted in red.Annotated RNA interaction motifs for Npl3.Npl3‐TAP is functional. npl3 cells are temperature sensitive at 37°C. This sensitivity is not observed in WT or Npl3‐TAP cells. Serial dilutions of indicated strains were assayed on YPD. Cells were plated at indicated temperatures and grown for 48 h.Npl3‐TAP association to telomeres does not change between telomerase‐positive and tlc1 cells when cells are propagated after dissection for 60 population doublings. Cross‐linked samples from indicated strains were used in a TAP‐ChIP. Enrichment at telomeres was determined by quantitative PCR on indicated telomeres. Data represent mean % input ± SEM relative to cells arrested in alpha factor *n* = 3 (unpaired *t*‐test, two‐tailed *P < 0.05, **P < 0.01). PD60 refers to 60 population doublings in the absence of TLC1 (telomerase RNA subunit).Npl3‐TAP protein levels do not change in tlc1 cells. Protein levels are determined using PAP (to detect the TAP tag) and anti‐actin antibodies for loading comparison.Data information: PAP, peroxidase anti‐peroxidase; PD, population doublings; TAP, tandem affinity purification tag.

To identify proteins associated with telomeric sequences in *Saccharomyces cerevisiae*, we performed quantitative label‐free interactomics. Using specific primer pairs, we synthesized DNA baits with a telomere‐mimetic sequence (telomere bait) and a non‐related DNA sequence (control bait). Oligonucleotides were polymerized using T4 DNA ligase and polynucleotide kinase. The resulting concatomer baits, largely double‐stranded, were subsequently biotinylated, immobilized on streptavidin‐coated beads, and incubated with protein extracts from wild‐type (WT) cells. Associated proteins were eluted and identified using quantitative proteomic methods (Fig 1A). We identified 69 proteins that specifically associated with the telomeric sequence *in vitro* (Fig 1B–D). Among them, we identified Rap1, the well‐characterized double‐stranded telomere binding protein in *S. cerevisiae* 26, validating our experimental approach. Gene Ontology (GO) analysis shows that the interactors are significantly enriched for nucleic acid binding, RNA binding, and DNA binding (Fig 1C). Surprisingly, 32 of the 69 proteins were annotated as RNA‐binding proteins (RBPs; Fig 1D, indicated with black boxes). Although the telomeric baits do not harbor RNA, we anticipate that TERRA, along with its telomeric binding factors that have been released into the lysate, is re‐associating to the bait. Alternatively, the RNA in the extracts may form RNA–DNA hybrids *in trans* 27, 28, 29 and recruit RNA and R‐loop interacting factors. Many of the identified RBPs have previously been demonstrated to interact with each other in complexes (Fig EV1A), suggestive of a functional role in telomere biology.

Although not the strongest interactor identified, we focus on the heterogeneous nuclear riboprotein (hnRNP)‐like protein Npl3 (Fig 1B) as it has been implicated in transcription and R‐loop regulation and harbors multiple RNA‐binding motifs (Fig EV1B) 30. Moreover, deletion of *NPL3* anticipates senescence onset in telomerase‐negative cells 31. Therefore, we hypothesized that Npl3 may be regulating rates of replicative senescence (senescence via telomere shortening) through regulation of the long non‐coding RNA (lncRNA), TERRA, at telomeres.

We validated the association of Npl3 to telomeres *in vivo* by performing chromatin immunoprecipitation (ChIP) of a functional TAP‐tagged version of *NPL3* (Npl3‐TAP) expressed under its native promoter (Fig EV1C). As Npl3 regulates senescence rates in telomerase‐negative cells, we tested whether it may associate preferentially to critically short telomeres. We verified that in telomerase‐negative cells (*tlc1*) critically short telomeres accumulate in senescent cells after 90 population doublings following telomerase loss, based on telomere length analysis on Southern blots and the reduced proliferation potential (Fig 1E and F). Subsequently, we characterized the binding of Npl3 to telomeres in telomerase‐negative cells using ChIP‐qPCR (Figs 1G and EV1D). In agreement with our interactomics data, we verified that Npl3 can associate with telomeres in telomerase‐positive cells, *in vivo* (Fig 1G). Npl3 was even further enriched in telomerase‐negative cells when critically short telomeres accumulate (Fig 1G, PD90) but not at short telomeres from cells that had undergone only 60 population doublings in the absence of telomerase (Fig EV1D). These data demonstrate that the increased Npl3 binding at critically short telomeres (PD90) is not simply due to the absence of *tlc1*, but rather a specific feature of deeply senescent cells (i.e., the accumulation of critically short telomeres). The stronger association of Npl3 to critically short telomeres (PD90) is not due to increased Npl3 protein levels in *tlc1* cells (Fig EV1E). In summary, we have identified Npl3 as a telomere binding protein in yeast and its association to telomeres increases when critically short telomere accumulates during replicative senescence.

### TERRA recruits Npl3 to telomeres

**Figure 2:**
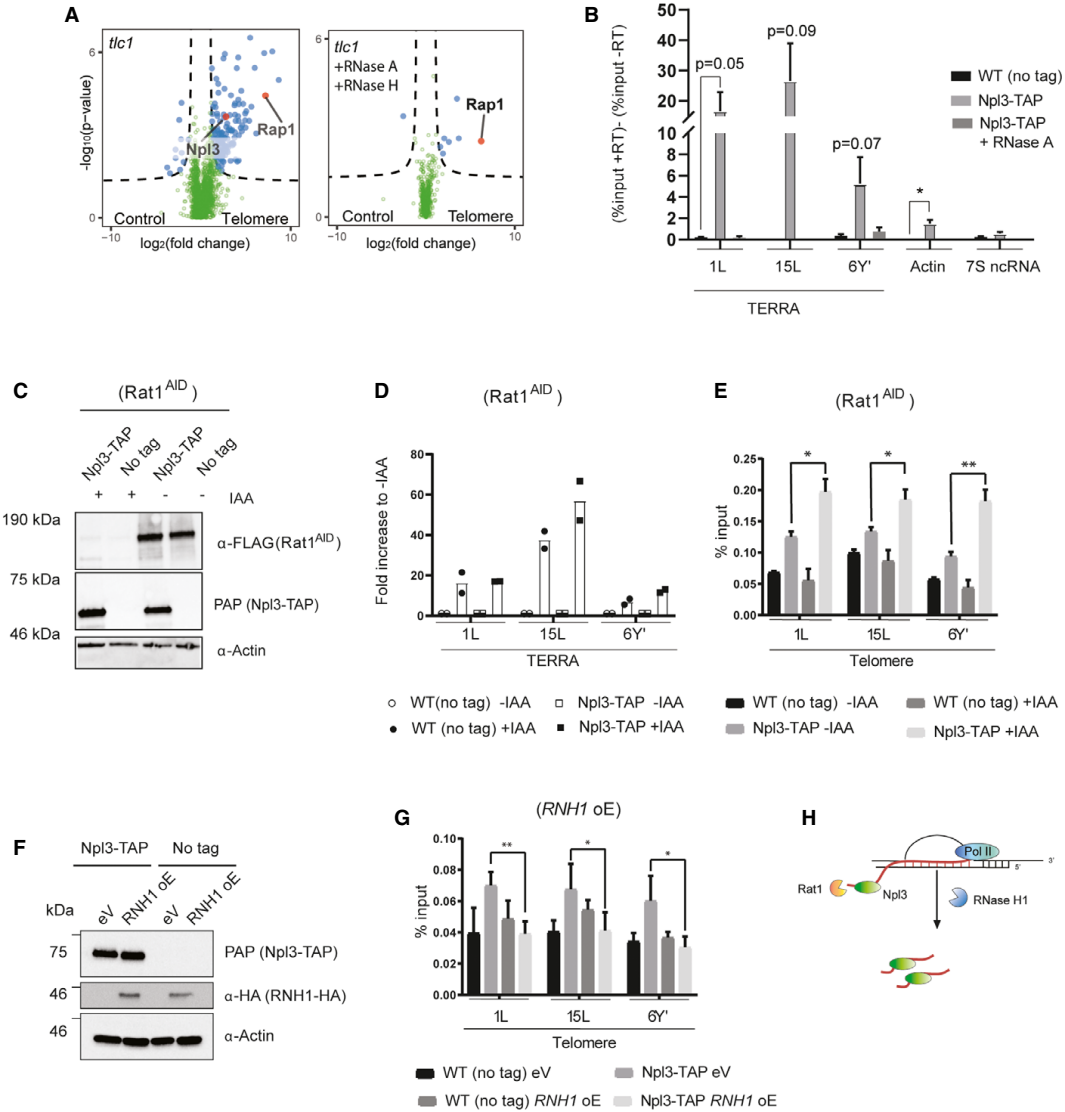
Npl3 associates with telomeres in a TERRA‐dependent manner ATelomere interactors were identified and plotted as a volcano plot as in Fig 1A and B and using tlc1 protein extracts. Interactors were identified using untreated protein extracts from tlc1 cells (left panel) or digested with RNase A and RNase H (right panel).BNpl3 associates with TERRA. Cross‐linked samples from the indicated strains were subjected to Npl3‐TAP RIP. Enrichment of the indicated RNAs was determined by quantitative PCR on pulled‐down reverse‐transcribed RNA. Data represent mean % input ± SEM *n* = 3 (unpaired *t*‐test, two‐tailed *P < 0.05, **P < 0.01).C–ENpl3 associates strongly with telomeres in the absence of Rat1. (C) The Rat1‐AID‐tagged protein is strongly degraded following 1 h of 1 mM IAA treatment. Protein levels are determined using anti‐FLAG (Rat1‐AID) and anti‐actin antibodies. (D) TERRA levels increase in cells expressing the Rat1‐AID variant after 1‐h treatment with 1 mM IAA. TERRA levels were determined by quantitative PCR on reverse‐transcribed RNAs after phenol–chloroform extraction. RNA levels are normalized to 7S ncRNA levels. Data represent fold increase to corresponding IAA samples *n* = 2. (E) Npl3‐TAP associates strongly with telomeres after 1 mM IAA treatment in cells with Rat1‐AID. Cross‐linked samples from indicated strains were used in a TAP‐ChIP. Enrichment at telomeres was determined by quantitative PCR on indicated telomeres. Data represent mean % input ± SEM *n* = 3 (paired *t*‐test, one‐tailed *P < 0.05, **P < 0.01).F, GNpl3 associates with telomeres in an R‐loop‐dependent manner. (E) RNH1 was overexpressed from a galactose‐inducible promoter. Protein levels are determined using anti‐HA and anti‐actin antibodies. (F) Npl3‐TAP associates with telomeres in an R‐loop‐dependent manner. Cross‐linked samples from indicated strains were used in a TAP‐ChIP. Enrichment at telomeres was determined by quantitative PCR on indicated telomeres. Data represent mean % input ± SEM *n* = 3 (paired *t*‐test, one‐tailed *P < 0.05, **P < 0.01).HNpl3 associates with TERRA at telomeres, likely through association with the nascent transcript. When Rat1 is absent, TERRA is stabilized and Npl3 would persist at telomeres bound to TERRA. RNaseH1 resolves telomeric RNA–DNA hybrids, which may release nascent TERRA with Npl3 bound.Data information: eV, empty vector control, oE, overexpression, IAA, indole‐3‐acetic acid, AID, auxin inducible degron. Individual *P*‐values are indicated in the figure.

**Figure EV2:**
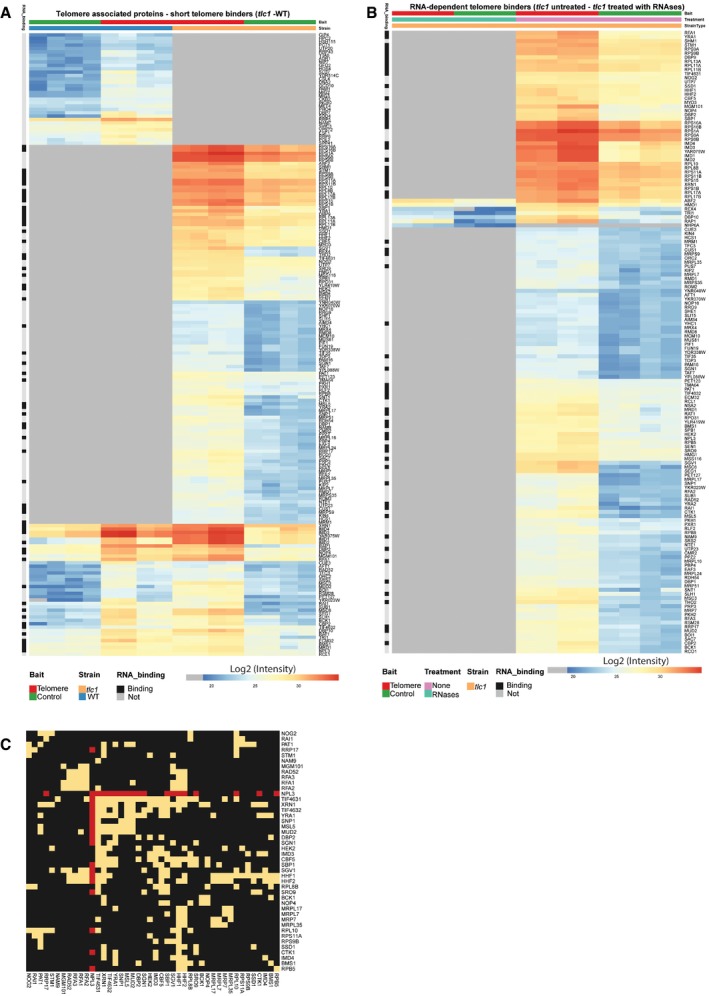
RNA‐dependent telomere‐associated proteins in yeast Heatmap for enriched proteins at the telomere bait in WT and tlc1 cells. Color code indicates measured intensities in the telomere pull‐down. Heatmap was constructed using the “pheatmap” package in R, where clustering is performed using complete data based on the Euclidean distance. Gray indicates not enriched.Heatmap for enriched proteins at the telomere bait tlc1 cells and tlc1 cells treated with RNAse A and RNase H. Color code indicates measured intensities in the telomere pull‐down. Heatmap was constructed using the “pheatmap” package in R, where clustering is performed using complete data based on the Euclidean distance. Gray indicates not enriched.RNA‐dependent telomere interactors in tlc1 cell lysates form protein interaction clusters. Proteins identified exclusively in tlc1 non‐treated pull‐downs when compared to tlc1 RNase A‐ and RNase H‐treated extracts were used for the analysis (i.e., the RNase sensitive interactors). Biogrid protein interactions are represented as a Heatmap. Proteins with less than five physical interactions were filtered out. Yellow is used for presence and black for absence of interaction. Clustering is performed using the complete data based on binary distance. Npl3 interactors are highlighted in red.

Npl3 associates more strongly to shortened telomeres *in vivo* (Fig 1G). Since short telomeres accumulate TERRA and telomeric R‐loops 15, 32, 33 and given that Npl3 is an RNA‐regulatory protein, we hypothesized that TERRA may mediate the association of Npl3 to short telomeres. We tested whether the association of Npl3 to telomeres is RNA‐mediated using quantitative interactomics by propagating telomerase‐negative cells for 90 population doublings and performing telomere pull‐downs as outlined in Fig 1A in the presence or absence of recombinant RNase A and RNase H. With this approach, we verified that Npl3 associates with telomeric baits in cells with short telomeres (Fig 2A, left panel) and show that this interaction is lost when the protein extracts are incubated with RNAse A and RNAse H (Fig 2A, right panel). We were also able to compare *in vitro* telomere interactors between telomerase‐positive and telomerase‐negative cell extracts (Fig EV2A). Strikingly, approximately 95% of the proteins identified in the *tlc1* telomere pull‐down no longer associate with the telomere baits upon treatment with RNase A and RNase H (Fig EV2B). Many of the RNase A/H sensitive telomere interactors form functional interactions among themselves (Fig EV2C). These results suggest that several proteins, and protein complexes, associate with short telomeres in an RNA‐dependent manner. We did not detect changes in the association of the ds telomeric DNA binding protein Rap1 to the telomeric bait upon RNase treatment, further validating our approach.

Our results demonstrate that Npl3 associates with telomeric sequences in an RNA‐dependent manner and suggest that elevated TERRA levels in *tlc1* cells promote the association of Npl3 to short telomeres. Npl3 is presumed to associate with many, if not all, RNAP II transcripts, and can bind to both ss and ds telomeric sequences (RNA and DNA) *in vitro* 30, 31. We tested the association of Npl3 to TERRA *in vivo* using RNA immunoprecipitation (RIP) followed by qPCR. Using TAP‐tagged Npl3, we pulled down soluble Npl3‐associated RNAs after protein–RNA cross‐link. To quantify the RNA binding, we reverse‐transcribed associated RNAs and performed qPCR on specific loci (Fig 2B). As expected, Npl3 binds to the highly transcribed *ACT1* (actin) mRNA (Fig 2B). Npl3 also associates with TERRA stemming from different telomeres *in vivo* (Fig 2B). Consistent with Npl3 associating preferentially with RNAP II transcripts, we did not detect 7S RNA, the RNA component of the signal recognition particle encoded by the *SCR1* gene, in the Npl3 RIP experiments (Fig 2B). Therefore, Npl3 associates with TERRA *in vivo*, suggesting that TERRA may be responsible for its association to telomeres.

To test this hypothesis, we investigated whether the binding of Npl3 to telomeres would be altered with varying TERRA and TERRA R‐loop levels. To increase TERRA levels, we performed Npl3‐TAP ChIP in a Rat1^AID^ (auxin inducible degron) harboring strain. Upon addition of 1 mM auxin, the Rat1^AID^ allele is rapidly degraded and TERRA levels increase (Fig 2C and D, 15, 18). In the presence of IAA (TERRA stabilization), Npl3 association to telomeres increases at all telomeres tested (Fig 2E), demonstrating that TERRA levels correlate with the amount of Npl3 at telomeres. Based on the fact that Npl3 associates with TERRA (Fig 2B), these data indicate that TERRA is responsible for Npl3 localization to telomeres.

To test whether R‐loops are involved in Npl3 recruitment to telomeres, we measured the Npl3 binding to telomeres by ChIP in cells overexpressing *RNH1* from a galactose‐inducible promoter (Fig 2F and G), which efficiently reduces telomeric R‐loops without disrupting total TERRA levels 15, 19, 33. The association of Npl3 to telomeres is reduced upon *RNH1* overexpression, supporting the notion that TERRA R‐loops promote the recruitment of Npl3 to telomeres (Fig 2G). In summary, Npl3 associates with telomeres in a TERRA‐ and R‐loop‐dependent manner. Based on Npl3's know function 30, 34, we propose that it associates with nascent TERRA transcripts at telomeres (Fig 2H) as it does for other RNAP II transcripts 30. The depletion of Rat1 would then result in TERRA stabilization and allow Npl3 to persist in the vicinity of telomeres hence accounting for its increase ChIP signal (Fig 2E). The resolution of R‐loops by *RNH1* overexpression would potentially release Npl3‐bound TERRA and account for the decreased ChIP signal of Npl3 at telomeres (Fig 2G).

### NPL3 stabilizes telomeric R‐loops

**Figure 3:**
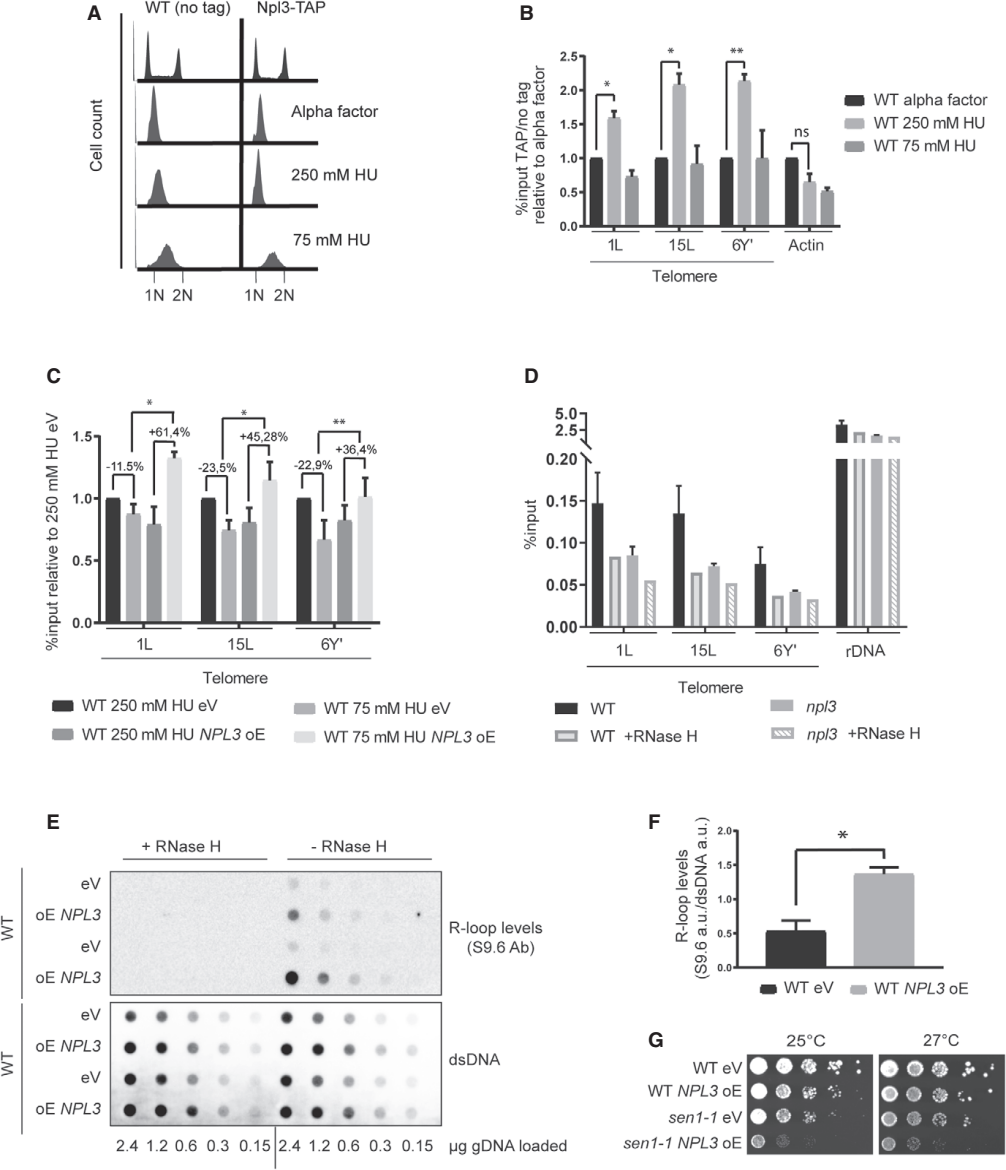
NPL3 stabilizes R‐loops A, BNpl3 associates with telomeres in early S. (A) Cell cycle profiles of indicated strains. Cells were synchronized in G1 with alpha factor for 2.5 h and released into the cell cycle at 30°C in pre‐warmed medium supplemented with the indicated HU concentrations. (B) Npl3‐TAP associates strongly with telomeres in early S (250 mM HU). Cross‐linked samples from indicated strains were used in a TAP‐ChIP. Enrichment at telomeres was determined by quantitative PCR on indicated telomeres. Data represent mean % input ± SEM relative to cells arrested in alpha factor *n* = 3 (paired *t*‐test, two‐tailed *P < 0.05, **P < 0.01).CNPL3 over expression stabilizes telomeric R‐loops. The indicated strains were grown on 1% raffinose 2% galactose to induce NPL3 over expression. Cross‐linked samples were subjected to DRIP. DNA‐RNA hybrids were immunoprecipitated using the S9.6 antibody. R‐loop levels were determined by quantitative PCR on indicated telomeres. Data represent mean % input ± SEM relative to 250 mM HU eV *n* = 3 (paired *t*‐test, two‐tailed *P < 0.05, **P < 0.01).DDeletion of NPL3 decreases telomeric R‐loops. The indicated strains were cross‐linked and subjected to DRIP. DNA‐RNA hybrids were immunoprecipitated using the S9.6 antibody. R‐loop levels were determined by quantitative PCR on indicated telomeres. Data represent mean % input ± SEM *n* = 3. Specificity of S9.6 antibody was determined treating a fraction of the samples with RNase H.E–GNPL3 stabilizes R‐loops. (E) R‐loop dot blot. The indicated strains were grown on 1% raffinose 2% galactose for 72 h and subsequently released into 1% raffinose 2% galactose or 2% glucose medium for six PDs. R‐loop levels and dsDNA levels were determined using the S9.6 antibody and anti‐dsDNA antibodies. Specificity of the S9.6 antibody was confirmed by treatment with RNase H. Loaded genomic DNA content is indicated at the bottom of the figure. (F) R‐loop dot blot quantification. Data represent R‐loop signal as the S9.6 antibody signal relative to corresponding dsDNA signal *n* = 3 (unpaired *t*‐test, two‐tailed *P < 0.05). (G) Serial dilutions of indicated strains were assayed on 1% raffinose 2% galactose media. Plates were imaged after 72 h incubation at indicated temperatures.Data information: ds, double‐strand; eV, empty vector control; HU, hydroxyurea; ns, not significant; oE, over expression. *P < 0.05, **P < 0.01.

TERRA and TERRA R‐loop levels are regulated during the cell cycle, with both showing an increase in early S phase and a decrease in late S phase 15, 35. To test whether Npl3 also associates with telomeres in a cell cycle‐regulated manner, similar to TERRA, we measured the binding of Npl3 to telomeres by ChIP‐qPCR in different cell cycle phases. Cells were arrested in G1 with alpha factor and released into medium containing either 250 or 75 mM HU to arrest the cells in early and late S phase, respectively, as previously described 15 (Fig 3A). We detected an increased association of Npl3 to telomeres in early S (250 mM HU) which declined in late S (75 mM HU; Fig 3B). Therefore, in terms of the cell cycle, the association of Npl3 to telomeres is regulated in an identical manner to TERRA and R‐loops 15. This is consistent with a TERRA/R‐loop‐dependent localization of Npl3 to telomeres.

As Npl3 has previously been implicated in the regulation of R‐loops 34, we tested whether or not R‐loop regulation at telomeres may be affected by the absence or presence of Npl3. First, we analyzed the cell cycle regulation of telomeric hybrids by DRIP (DNA‐RNA IP) in cells that overexpressed *NPL3* under the control of a strong galactose‐inducible promoter (Fig 3C). In empty vector (eV) control cells, the telomeric R‐loop levels decrease in late S compared to early S at all telomeres, as it has been previously reported (Fig 3C, 15). However, upon overexpression of *NPL3*, the telomeric R‐loops do not get removed in a timely manner and even increase in abundance (Fig 3C). We conclude that high levels of Npl3 can prevent the cell cycle‐regulated R‐loop removal at telomeres. Consistently, in the absence of Npl3 (*npl3* cells) hybrid abundance is strongly reduced at telomeres (Fig 3D), whereas the largely RNAP I transcribed rDNA locus was unaffected in terms of Npl3 sensitive hybrids. In summary, these data reveal that Npl3 has the ability to stabilize R‐loops at telomeres.

To determine whether *NPL3* may also stabilize RNA–DNA hybrids in a general manner, or whether this was a telomere‐specific phenomenon, we overexpressed *NPL3* and analyzed RNA–DNA hybrid levels via Southwestern blotting (dot blot) with the S9.6 antibody 36 that recognizes RNA–DNA hybrids (Fig 3E). To exclude unspecific binding of the S9.6 antibody, we treated the genomic DNA with RNase III and T1 either in the presence, or absence, of recombinant RNase H. These data revealed that *NPL3* overexpression increases global RNA–DNA hybrid levels approximately threefold over eV control cells (Fig 3E and F). We hypothesized that if Npl3 stabilizes R‐loops, then the overexpression of *NPL3* should further impair the viability of mutants that already accumulate toxic R‐loops, such as *sen1‐1* mutants 37, 38. Indeed, the overexpression of *NPL3* in *sen1‐1* mutants inhibits the growth of the mutant cells but not WT cells (Fig 3G). It is interesting that the overexpression of *NPL3* does not lead to toxicity in WT cells, where R‐loops are also present, but only in *sen1‐1* cells where R‐loop stability is further increased. This scenario may be a reflection of what is occurring at critically short telomeres, where R‐loops become stabilized in order to promote HDR 14, 15. Indeed, Npl3 specifically accumulates at short telomeres (Fig 1G). Taken together, Npl3 appears to play a critical role with respect to R‐loop regulation. On the one hand, as previously published, it can prevent R‐loop accumulation by binding the nascent transcript and thereby promoting RNA export 30. On the other hand, we now observe that Npl3 can promote hybrid stability when R‐loops persist. How can such paradoxical roles for Npl3 be explained? When an R‐loop becomes stabilized, one of the negative consequences is transcription stalling 39, which would result in proteins associated with the nascent transcript (including Npl3) becoming “trapped” in the vicinity of the paused polymerase. The accumulation of proteins bound to the nascent transcript may have the effect of preventing access of RNA–DNA hybrid removal factors. In this respect, Npl3 behaves similar to other yeast hnRNP‐like protein, Yra1, that can also regulate R‐loop levels in two opposing manners 33, 40.

### NPL3 regulates telomeric R‐loops to prevent premature senescence

**Figure 4:**
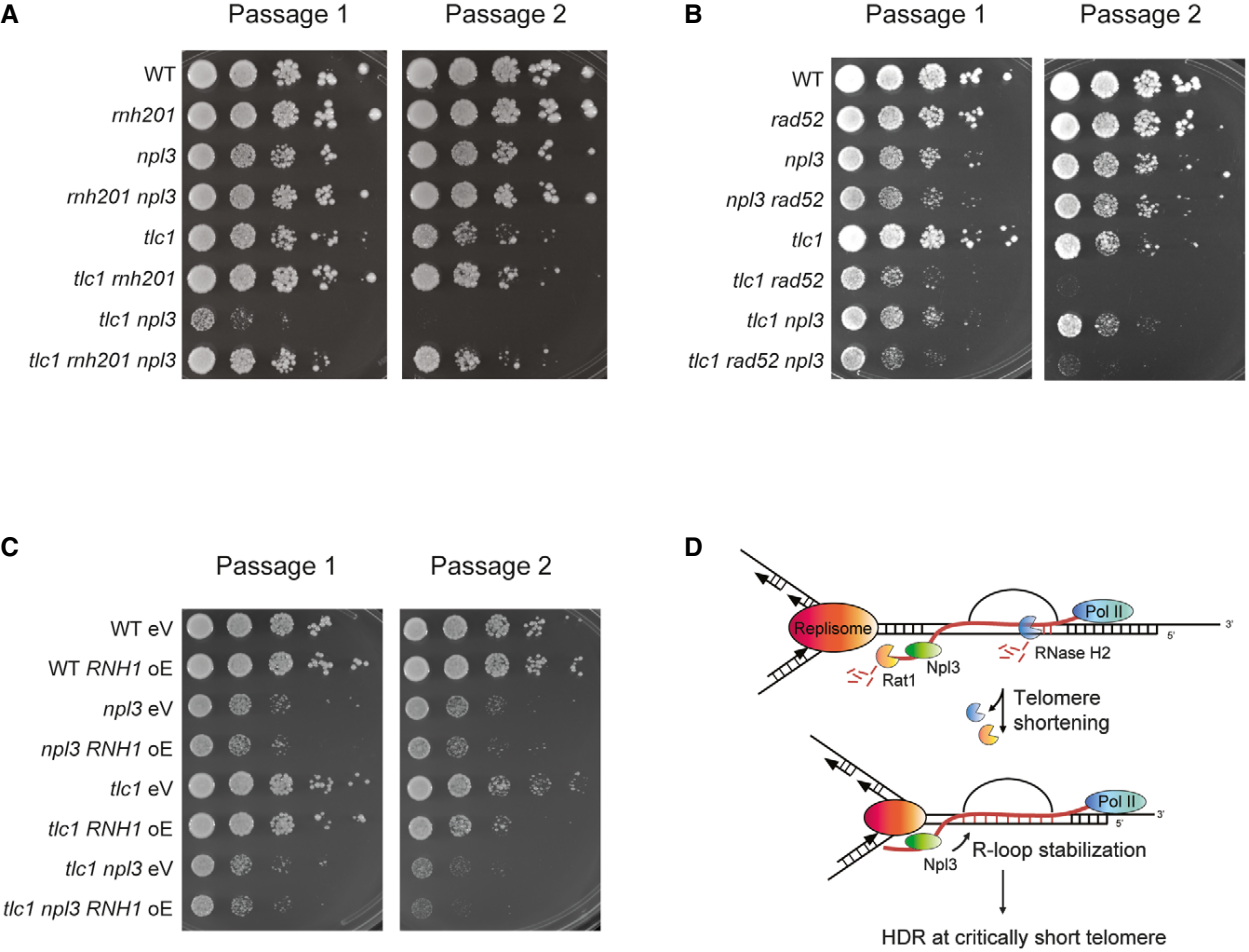
NPL3 stabilizes telomeric R‐loops to counteract accelerated senescence Serial dilutions of indicated strains were assayed on YPD media. Cells were plated after 30–50 generations propagated on YPD agar media. Two consecutive passages from one representative biological replicate are shown. Plates were imaged after 72 h growth at 30°C.Serial dilutions of indicated strains were assayed on YPD media. Cells were plated after 30–50 generations propagated on YPD agar media. Two consecutive passages from one representative biological replicate are shown. Plates were imaged after 48 h growth at 30°C.Serial dilutions of indicated strains were assayed on SC‐His media. Cells were plated after 30–50 generations propagated on SC‐His agar media. Two consecutive passages from one representative biological replicate are shown. Plates were imaged after 72 h growth at 30°C.Proposed model for Npl3 function at telomeres. At normal length telomeres, Npl3 associates transiently with telomeres, likely through interaction with the TERRA nascent transcript. As TERRA and R‐loops are removed in late S phase, Npl3 is also lost. When telomeres shorten, increased levels of telomeric R‐loops and TERRA are a result of decreased Rat1 and RNase H2 localization to telomeres. This has the effect of allowing Npl3 to remain associated with telomeres. The continued presence of Npl3 further promotes R‐loop stability, potentially by preventing access of RNA–DNA hybrid removal factors. The continued presence of stable R‐loops at telomeres promotes HDR at the short telomeres, likely upon interaction with the oncoming replisome, which in turn prevents the early onset of replicative senescence.

**Figure EV3:**
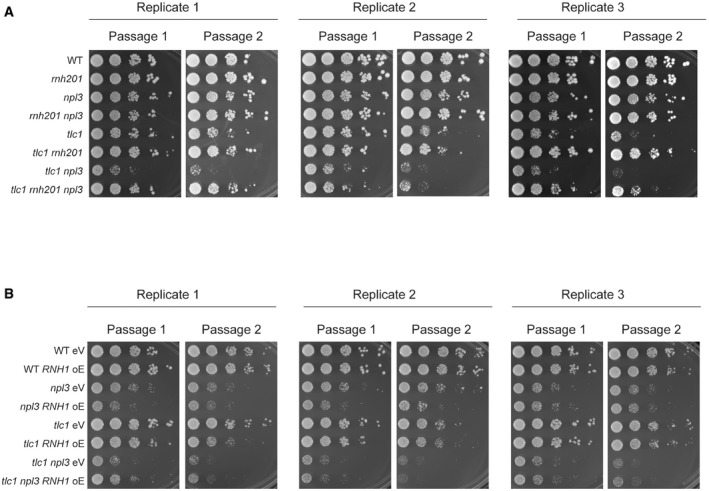
Npl3 regulates senescence rate Serial dilutions of indicated strains were assayed on YPD media. Cells were plated after 30–50 generations propagated on YPD agar media. Two consecutive passages from 3 different biological replicates are shown. Plates were imaged after 72 h growth at 30°C.Serial dilutions of indicated strains were assayed on SC‐His media. Cells were plated after 30–50 generations propagated on SC‐His agar media. Two consecutive passages from three different biological replicates are shown. Plates were imaged after 72 h growth at 30°C.

Previous studies have shown that the deletion of *NPL3* accelerates senescence rates in the absence of telomerase 31. Based on our results, that Npl3 associates with short telomeres (Fig 1) and can stabilize telomeric R‐loops (Fig 3C and D), we hypothesize that R‐loop instability, and hence faulty HDR of critically short telomeres, may be responsible for the enhanced senescence rates. To study the functional role of Npl3 at short telomeres, we performed senescence assays by propagating different yeast strains over consecutive passages on agar plates in the presence and absence of telomerase and in different genetic settings (Fig 4). Deletion of *RNH201* in telomerase‐negative cells strongly delays senescence onset, due to the accumulation of TERRA R‐loops and increased rates of telomeric HDR 15, 19 (Fig 4A). Importantly, the deletion of *RNH201,* and hence the stabilization of telomeric R‐loops, rescues the accelerated senescence rate of *tlc1 npl3* cells (Figs 4A and EV3A, bottom two rows), thereby providing genetic evidence that a lack of R‐loops may be responsible for the senescence defects in *tlc1 npl3* cells. Additionally, we compared the senescence rate of telomerase‐negative cells impaired for homologous recombination (*rad52* cells) in the presence and absence of *NPL3* (Fig 4B). Consistent with a role for Npl3 promoting HDR, we did not detect additive effects in terms of increased senescence rates upon deletion of *RAD52* and *NPL3* together in *tlc1* cells as compared to the single deletions. Furthermore, in telomerase‐negative cells, the overexpression of *RNH1* accelerates senescence onset due to the degradation of TERRA R‐loops 19 (Figs 4C and EV3B, rows 5 and 6). As with the deletion of *RAD52*, the overexpression of *RNH1* does not appear to increase the rate of replicative senescence in telomerase‐negative *NPL3* deletions (Figs 4C and EV3B, bottom two rows), consistent with decreased R‐loops being responsible for the early onset senescence in *NPL3‐*deleted cells.

Taken together, these data extend our knowledge regarding how R‐loops are regulated at telomeres and how they help to govern rates of replicative senescence (Fig 4D). When telomeres are “normal length”, RNase H2 and Rat1 prevent TERRA and R‐loop accumulation 15, likely to avoid transcription–replication conflicts and hence promote telomere stability. At normal length telomeres, Npl3 association is restricted to the early S phase of the cell cycle. In this condition, Npl3 likely associates with the nascent TERRA transcript, but gets eventually removed as TERRA and TERRA R‐loops are rapidly degraded by Rat1 and RNase H2, respectively (Fig 4D, top). At short telomeres, TERRA and R‐loop stabilization may result in transcription stalling and hence prolonged association of Npl3 to nascent TERRA. Npl3 bound to nascent TERRA may contribute to R‐loop stabilization by preventing the accessibility of R‐loop removal factors such as RNase H1 or Sen1. Stabilized R‐loops promote HDR when encountered by the replisome, which could result in an elongation event at critically short telomeres. In the absence of *NPL3*, R‐loops at telomeres are less stable, as they might be more accessible to R‐loop degrading enzymes. In this scenario, the absence of stable R‐loops may anticipate senescence onset in *tlc1 npl3* cells similar to what has been previously shown when R‐loops are depleted through *RNH1* overexpression.

## Materials and Methods

### Yeast strains and cultures

The strains used on this study are listed in Table EV1.Yeast culture was conducted at 30°C in YPD medium unless otherwise indicated. Primers and plasmids used in this study are listed in Tables EV2 and EV3. Antibodies used in this study are listed in Table EV4.

### Spotting assay

Cells were grown over night at appropriate temperature and media. Saturated cell cultures were diluted to OD_600_ = 0.5 and spotted in tenfold dilutions into corresponding agar plates. Plates were incubated at the indicated temperatures and time and imaged using Bio‐Rad ChemiDoc Touch Imaging System.

### Senescence assays

Senescence rates were determined as previously described in liquid media 15, 19. Briefly, heterozygous diploid strains were dissected in corresponding media (agar plates) and haploid progeny was incubated for 72 h at 30°C before scoring haploid genotypes. Cells were diluted to OD_600_ = 0.01 every 24 h for 12 days. OD_600_ was recorded every 24 h as a measurement of cell growth before diluting cells. %viability is determined as the relative OD_600_ to the first day of measurement (considered as 100% viability). Population doublings were calculated every day as the log_2_(OD_600_/0.01). Thirty–forty additional population doublings were considered for the initial cell growth after dissection of heterozygous diploid strains. Graphs were plotted using Prism 8.

*tlc1 npl3* cells senesce rapidly, so senescence rate was determined by propagating cells in solid agar plates using the spotting assay. Heterozygous diploid strains were dissected in corresponding media (agar plates). Haploid progeny was incubated for 72 h at 30°C before scoring haploid genotypes. It is important that *npl3* cells are not stored at 4°C as this strain is cold sensitive. Cells were then resuspended in corresponding liquid media. Cells were diluted to OD_600 _= 0.5 and spotted in tenfold dilutions into corresponding agar plates (passage 1). Plates were incubated at the indicated temperatures and time and imaged using Bio‐Rad ChemiDoc Touch Imaging System. After incubation, grown cells from the spotting plate were again resuspended in liquid media, diluted to OD_600 _= 0.5, and spotted in tenfold dilutions into corresponding agar plates (passage 2). Plates were incubated at the indicated temperatures and time and imaged using Bio‐Rad ChemiDoc Touch Imaging System. The diploid strains yLP130 and yLP131 were transformed with the plasmids pBL335 and pBL336 (respectively) before dissecting them on selective plates.

### Protein extraction for MS/MS

One hundred milliliter of exponentially growing cultures was collected by centrifugation for 3 min at 1,731 g‐force. Cell pellets were lysed in lysis buffer [50 mM Tris–HCl pH 7.5, 150 mM NaCl, 5 mM MgCl_2_, 1 mM PMSF supplemented with complete protease inhibitor cocktail (Roche)] by two rounds of 30 s at 6.5 M/s on a FastPrep machine (MP Biomedical). Cell extracts were resuspended in lysis buffer supplemented with 0.2% IGEPAL CA‐630 and centrifuged at 13,000 g‐force for 15 min at 4°C. The soluble fraction was subjected to a second round of centrifugation recovering the supernatant (soluble protein extract). Five hundred microgram of protein extract was used for each telomere pull‐down.

### Polymerization of DNA baits

Twenty‐five microgram of forward and reverse oligonucleotides harboring telomere and control sequence were diluted in annealing buffer (20 mM Tris–HCl, 10 mM MgCl_2_, 100 mM KCl) and denatured at 95°C for 10 min. Oligos were annealed by cooling down to room temperature and polymerized with 50 units of T4 Polynucleotide Kinase (NEB) for 2 h at 37°C. Fragments were ligated with 80 units of T4 DNA ligase (Thermo Scientific) at RT overnight. Ligated DNA baits were purified with phenol/chloroform extraction and biotinylated with N6‐(6‐Amino)hexyl‐dATP—Biotin (Biotin‐7‐dATP; Jena Bioscience) and 30 units of DNA polymerase Klenow fragment exo‐ (Thermo Scientific). DNA baits were purified using Microspin Sephadex G50 columns (GE Healthcare) before performing the telomere pull‐down.

### Telomere pull‐down

Biotinylated DNA baits were immobilized on magnetic streptavidin beads (MyOne C1 Streptavidin Dynabeads, Thermo) for 30 min at room temperature on a rotation wheel. DNA baits were incubated with 500 μg of protein extracts diluted in PBB buffer (150 mM NaCl, 50 mM Tris–HCl pH 7.5, 0.5% IGEPAL CA‐630, 5 mM MgCl_2_, 1 mM DTT) using 20 μg sheared salmon sperm DNA as a competitor (Thermo). Protein extracts were incubated with DNA baits for 1 h at 4°C on a rotation wheel. A fraction of the samples was treated with five units of RNase H (NEB) and 20 μg of RNase A (Thermo Scientific) during incubation. DNA baits were washed three times with PBB buffer, and bound proteins were eluted by heating for 10 min at 75°C in 1× NuPAGE LDS buffer (Thermo) supplemented with 100 mM DTT.

### MS sample processing

Eluted proteins from telomere pull‐down were separated on a 4–12% NuPAGE Bis–Tris pre‐casted PAGE gel (Thermo). Eluates were run at 180V for 10 min and processed by in‐gel digestion 41. Briefly, samples were reduced in reduction buffer [10 mM DTT in 50 mM ammonium bicarbonate (ABC) buffer] for 1 h at 56°C and alkylated in alkylation buffer [50 mM iodoacetamide (IAA) in 50 mM ABC buffer] for 45 min in the dark. Proteins were digested with 2 μg protease LysC (Wako Chemicals) overnight at 37°C in 50 mM ABC buffer. Digested peptides were desalted on a C18 StageTip as described 42 and analyzed by nanoflow liquid chromatography on an EASY‐nLC 1000 system (Thermo) coupled to a Q Exactive Plus mass spectrometer (Thermo). The peptides were separated on a self‐packed reverse phase capillary (75 μm diameter, 25 cm length packed with C18 beads of 1.9 μm; Dr Maisch GmbH). The capillary was clamped on an electrospray ion source (Nanospray Flex™; Thermo). A 90 min gradient starting from 2 to 60% gradient acetonitrile in 0.1% formic acid was used at a flow of 225 nl/min. Data were collected in data‐dependent acquisition mode with one MS full scan followed by up to 10 MS/MS scan with HCD fragmentation.

### MS data processing and bioinformatic analysis

MS raw files were processed using the MaxQuant software (version 1.5.2.8) and the ENSEMBL *S. cerevisiae* protein database (Saccharomyces_cerevisiae.R64‐1‐1.24). LFQ quantitation and match between run options were activated. MaxQuant output files were analyzed using an in‐house R script. Briefly, known contaminants, reverse hits, and protein groups only identified by site were removed. Identified protein groups (minimum two peptides, one of them unique) were further filtered to a minimum of two quantification events per experiment. Missing values were imputed using a downshifted and compressed beta distribution within the 0.001 and 0.015 percentile of the measured values for each replicate individually. The LFQ intensities were log_2_‐transformed, and a two sample Welch *t*‐test was performed. Volcano plots were generated by plotting −log_10_(*P*‐values) and fold changes. The threshold line for enriched proteins is defined empirically with *P*‐value = 0.05, *s*0 = 1 and *c* = 0.5. Gene Ontology analysis for telomere‐associated candidates was performed with the PantherDB.org overrepresentation test (Release 20190701) with the annotation database released on 20190202. Fisher's exact test followed by Bonferroni correction was applied. Heatmaps for enriched proteins were generated using the “pheatmap” package in R with clustering the complete data based on the Euclidean distance. Biogrid protein interaction was clustered using the complete data based on binary distance.

### TAP‐ChIP, TAP‐RIP, and DRIP

One hundred–one hundred fifty milliliter of exponentially growing cultures was cross‐linked for 10 min with 1.2% formaldehyde (Applichem) after equal normalization of cell number. Samples were quenched with glycine (360 mM, Applichem) for 5 min at room temperature. After cooling down to 4°C on ice for 15 min, cells were pelleted at 4°C by centrifugation (1,731 rcf, 3 min), washed twice with ice‐cold PBS, and stored at −80°C until processing. Cell pellets were lysed in FA buffer (50 mM HEPES‐KOH pH 7.5, 140 mM NaCl, 1 mM EDTA pH8, 1% Triton X‐100, protease inhibitor cocktail) via 2 × 30 s rounds of 6.5 M/s FastPrep (MP Biomedical). Samples were diluted in FA buffer supplemented with 0.1% sodium‐deoxycholate (SOD). Chromatin extracts were separated by centrifugation (7 min at 17,949 g‐force) and then sonicated in two rounds of 10 cycles (30 s on, 30 s off) using the Bioruptor Pico (Diagenode). Sonication was verified by running 50 μl of extracts on 1% agarose gel after de cross‐linking overnight at 65°C, and digestion with Proteinase K (0.75 mg/ml, QIAGEN) and 20 μg of RNase A (Thermo Scientific). One milligram of sonicated chromatin extracts was incubated overnight at 4°C with 50 μl of pre‐washed IgG Sepharose beads (GE Healthcare) with 5% BSA. Fifty microliter of extracts was separated as an input control. For DRIP 1 mg of sonicated chromatin extracts were pre‐cleared with 30 μl of pre‐washed protein A Sepharose beads (GEHealthcare) for 1 h at 4°C and subsequently incubated with 2 mg S9.6 antibody (Kerafast) at 4°C for 1 h. After incubation, 50 μl of pre‐washed protein A Sepharose beads (GE Healthcare) were added to chromatin extracts and incubated overnight at 4°C. A fraction of the sample was treated with recombinant 50 units of RNase H (NEB) to test specificity of the S9.6 antibody. For TAP‐RIP, 2 mg of soluble extracts were incubated overnight at 4°C with 75 μl of pre‐washed IgG beads (GE Healthcare) with 5% BSA. Fifty microliter of extracts was separated as an input control. Beads were washed with 1 ml of FA buffer, buffer 500 (50 mM HEPES‐KOH pH 7.5, 500 mM NaCl, 1 mM EDTA pH8, 1% Triton X‐100, 0.1% SOD), buffer III (10 mM Tris–HCl pH 8, 1 mM EDTA pH 8, 150 mM LiCl, 1% NP40, 1% SOD), and TE buffer (100 mM Tris–HCl pH 8, 50 mM EDTA pH 8) at 4°C with 5 min incubation times between washes. For ChIP and DRIP, proteins were eluted in Elution buffer (50 mM Tris–HCl pH 7.5, 1% SDS, 10 mM EDTA pH8) in 2 × 8 min denaturation runs at 65°C. DNA was digested overnight at 65°C with 0.75 mg/ml proteinase K in Elution buffer. Digested DNA was purified using the PCR purification kit (QIAquick, QIAGEN) and eluted in 50 μl ddH_2_O. Two microliter of purified DNA was used for ChIP quantification by qPCR. For RIP, proteins were eluted in Elution buffer (50 mM Tris–HCl pH 7.5, 1% SDS, 10 mM EDTA pH 8) in 2 × 8 min denaturation runs at 65°C. DNA was de‐cross‐linked for 2 h at 65°C and subsequently digested with three units of DNAse I (QIAGEN) for 2 h at 37°C. After digestion, eluted samples were digested with proteinase K (0.75 mg/ml) for 2 h at 65°C. RNA samples were purified using the RNeasy MinElute Cleanup kit (QIAGEN). Purified RNA samples were digested once more with three units of DNAse I (QIAGEN) and purified. RNA samples were subjected to reverse transcription before quantification by qPCR for different loci. To test for IP specificity, one‐third of the eluted samples were digested with 20 μg of RNase A at 37°C for 30 min before reverse transcription. For reverse transcription, RNA samples were split into three reactions. One reaction contained the RIP eluted RNA, another contained the eluted RNA digested with RNase A. The last reaction was used as a negative control of reverse transcription. The RNA was incubated at 90°C for 1 min with 0,4 μl 25 mM dNTPs, 1 μl 10 μM oBL207, 0.4 μl 10 μM oBL293, and 0.4 μl 10 μM oMG60 in 10 μl final volume reaction. The RNA was then cooled down to 55°C at a 0.8°C/s temperature rate. A mix of 1 μl 100 mM DTT and 1 μl SuperScript III in 1× FS‐buffer (Invitrogen) was added to the reactions. Negative control sample did not contain SuperScript III reverse transcriptase. The RNA was reverse‐transcribed for 60 min at 55°C. The enzyme was inactivated at 70°C for 15 min. RNA samples were diluted with 30 μl H_2_O and subjected to qPCR.

### Dot blot

Cells were grown in 25 ml cultures and collected at exponential growth in appropriate medium. Genomic DNA was extracted using the Gentra Puregene Yeast/Bacteria Extraction kit (QIAGEN). 4.8 μg of DNA was digested for 2.5 h at 37°C with five units of RNase III (Ambion, Thermo Scientific) and one unit of RNAse T1 (Thermo Fisher). A fraction of the samples were additionally treated with 10 units of RNase H (NEB) for 2.5 h at 37°C. Digested DNA was split into two and spotted onto positively charged nylon membrane (Roche) in SSC 2× serial dilutions (1:2). Once dried, the membranes were cross‐linked with UV 30 s on auto cross‐link (1,200u Joules) and blocked with 5% milk in PBS‐0.1% Tween for 1 h at room temperature. Blocked membranes were incubated over night at 4°C in agitation with S9.6 antibody (Kerafast ENH001, 2 μg diluted in 3% BSA) and anti‐dsDNA antibody (Abcam ab27156, 1:1,000). Membranes were washed three times with PBS‐Tween 0.1% at room temperature and incubated with secondary antibody (Goat anti‐mouse, BioRad 170‐5047 1:3,000 in 5% milk in PBS‐0.1% Tween) for 1 h at room temperature. Membranes were developed using 10 ml of Super Signal West Pico Chemiluminescent Substrate (Thermo Scientific) in ChemiDoc Touch Imaging System (BioRad). Spot signal was quantified using ImageJ Software. Background was subtracted from S9.6 and dsDNA spots. S9.6 signal was normalized to dsDNA signal.

### Western Blot

1–2 OD_600_ units of exponentially growing cells were pelleted and resuspended in 150 μl of solution 1 (1.85 M NaOH supplemented with 1.09 M 2‐mercaptoethanol). After 10 min incubation on ice, 150 μl solution 2 [50% Trichloroacetic acid (TCA) in H_2_O] were added and further incubated 10 min on ice. Proteins were pelleted by centrifugation at 17,949 rcf for 2 min at 4°C and washed with 100% Acetone. Proteins were resuspended in 100–150 μl urea buffer (120 mM Tris–HCl pH 6.8, 5% glycerol, 8 M urea, 143 mM 2‐mercaptoethanol, 8% SDS, bromophenol blue) and denatured for 10 min at 65°C. Samples were loaded on 4–15% gradient pre‐casted polyacrylamide gels (BioRad) for 30 min at 200V and transferred to a nitrocellulose membrane using the Trans‐Blot Turbo (BioRad) on High molecular Weight Program. Membranes were blocked with 5% milk for 1 h at room temperature and incubated with the following antibodies: anti‐FLAG (Sigma Aldrich, F3165, mouse, 1:1,000), PAP (Sigma Aldrich, P1291, rabbit, 1:200), anti‐actin (Millipore, MAB1501R, mouse, 1:2,000), and anti‐HA (Covance, MMS‐101P, mouse, 1:2,000). After incubation with corresponding secondary antibody (Goat anti‐mouse, BioRad, 170‐5047, 1:3,000), proteins were imaged using Super Signal West Pico Chemiluminescent Substrate (Thermo Fisher) and ChemiDoc Touch Imaging System (BioRad).

### Flow cytometry

0.2 units of exponentially growing cells were collected for DNA content analysis. Cells were pelleted and fixed in 70% EtOH over night at 4°C. Fixed cells were treated with RNase A (0.25 mg/ml) in 50 mM Tris buffer pH 7.5 for 3 h at 37°C and subsequently treated with proteinase K (1 mg/ml) for 2 h at 50°C. Cells were sonicated with BRANSON sonifier 450 for at least 10 s (Constant mode) and diluted in 50 mM Tris–HCl pH 7.5 supplemented with SYTOX Green (Thermo Fisher Scientific). DNA content of at least 10,000 cells was analyzed by flow cytometry using a BD FACSVerse flow cytometer. Data analysis was performed with FlowJo (v10.5.3).

### Southern Blot

Exponentially growing cells were collected for genomic DNA extraction. Cells were lysed with a 900 mM sorbitol, 100 mM EDTA pH 8 solution supplemented with 14 mM 2‐mercaptoethanol, and five units of 100T lyticase (Sigma Aldrich). Spheroblasts were pelleted by centrifugation (17,949,956 g‐force 1 min) and resuspended in TE buffer. A solution containing 2.5 mM EDTA pH 8, 222 mM Tris‐base, and 2.2% SDS was added. Samples were incubated 30 min at 65°C before 80 μl 5 M potassium acetate were added. Samples were cooled down to 4°C for 1 h. After centrifugation (20,817 g‐force 15 min), soluble DNA was precipitated with ice‐cold 100% ethanol. DNA pellets were resuspended in TE buffer, and remaining RNA was digested with 25 μg RNAse A 60 min at 37°C (Thermo Fisher). DNA was precipitated with ice‐cold 100% isopropanol and resuspended in TE buffer. Five–ten microgram of extracted DNA was digested with 1 μl XhoI (NEB) for 5 h at 37°C and then loaded into a 0.8% agarose gel. DNA fragments were separated by electrophoresis at 50V overnight. The agarose gel was denatured for 1 h in denaturing solution (400 mM NaOH, 600 mM NaCl) and neutralized neutralizing solution (1 M Trizma base, 1.5 M NaCl pH 7.4) for 1 h. After neutralization, the DNA fragments were capillary transferred to a nylon membrane (Roche) in 10× SSC for 72 h and cross‐linked with UV light 30 s on auto cross‐link (1,200u Joules). The membrane was then incubated in hybridization solution (Perfect Hyb‐buffer, Sigma Aldrich) for 5 h at 55°C on rotation. The membrane was hybridized overnight at 55°C with a telomere‐specific probe generated by radioactive labeling with dATP alpha‐P^32^ (Perkin Elmer). After hybridization, the membrane was washed twice (1 h each) in washing solution I (2× SSC, 0.1% SDS) and twice with solution II (0.5× SSC, 0.1% SDS) both pre‐warmed to 55°C. The membrane was dried at room temperature for 30 min and exposed for 2–4 days. Membrane was imaged using Typhoon FLA 9500 (GE Healthcare).

### TERRA levels

Exponentially growing cells were collected and resuspended in 400 μl AE buffer (50 mM Sodium Citrate in 10 mM EDTA pH5.3) and lysed with 500 μl calibrated phenol (with AE buffer) at 65°C for 5 min. Aqueous phase was separated by centrifugation and mixed with 500 μl phenol–chloroform–isoamyl alcohol 5 min at room temperature. Aqueous phase was again separated by centrifugation and separated for RNA precipitation. RNA was precipitated with 40 μl 3 M sodium acetate and 1 ml 100% ethanol. RNA was pelleted and washed with 80% ethanol. Air‐dried pellet was subsequently resuspended in a solution containing 3 μl DNAse I (QIAGEN) in RDD buffer to digest genomic DNA. DNA was digested for 45 min at 37°C, and remaining RNA was purified with RNeasy MinElute Cleanup kit (QIAGEN). Fifty microgram of RNA was purified and digested twice more with DNAse I. The RNA was incubated at 90°C for 1 min with 0.4 μl 25 mM dNTPs, 1 μl 10 μM oBL207, and 0.4 μl 10 μM oMG60 in 10 μl final volume reaction. The RNA was then cooled down to 55°C at a 0.8°C/s temperature rate. A mix of 1 μl 100 mM DTT and 1 μl SuperScript III in 1× FS‐buffer (Invitrogen) was added to the reactions. Negative control sample did not contain SuperScript III reverse transcriptase. The RNA was reverse‐transcribed for 60 min at 55°C. The enzyme was inactivated at 70°C for 15 min. RNA samples were diluted with 30 μl H_2_O and subjected to qPCR.

## Author contributions

Conceptualization: BL, FB, LP‐M; Methodology: BL, FB, LP‐M, MO; Investigation, LP‐M, MO; Writing—original draft: BL, FB, LP‐M; Funding acquisition: BL, FB; Supervision: BL and FB.

## Conflict of interest

The authors declare that they have no conflict of interest.

## Supporting information



Expanded View Figures PDF

Table EV1

Table EV2

Table EV3

Table EV4

Review Process File

## Data Availability

The mass spectrometry proteomics data have been deposited to the ProteomeXchange Consortium via the PRIDE partner repository with the dataset identifier PXD016772 (https://www.ebi.ac.uk/pride/archive/projects/PXD016772).
