# Supplementary material for: Npl3 stabilizes R‐loops at telomeres to prevent accelerated replicative senescence
Source: EMBO Rep. 2020 Feb 6;21(3):e49087. doi: 10.15252/embr.201949087 (PMC7054685; doi:10.15252/embr.201949087)
Supplement: Supplementary file 1 — Expanded View Figures PDF [file EMBR-21-e49087-s001.pdf]

Expanded View Figures

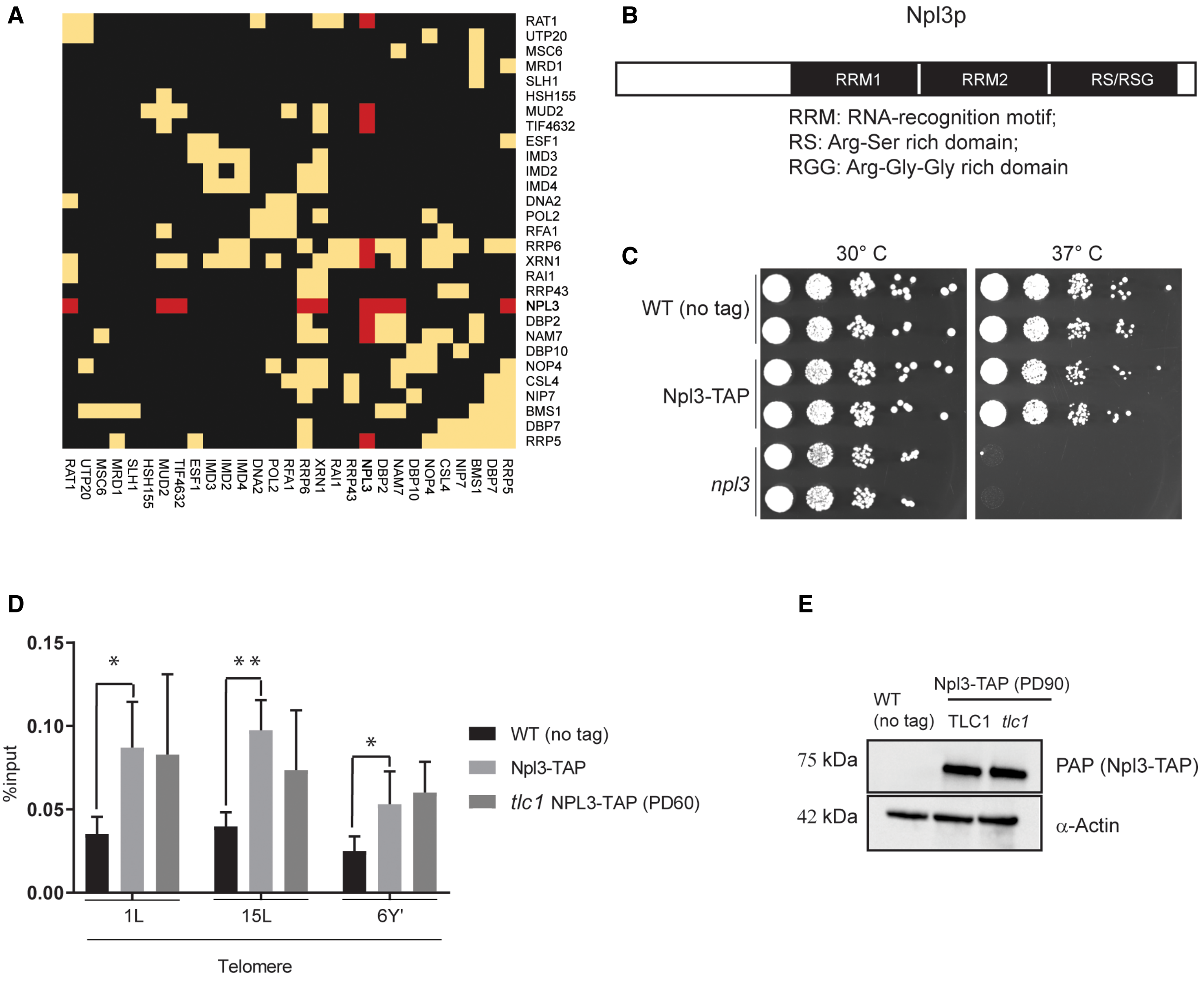

Figure EV1. A screen for telomere-associated proteins in yeast.

A RBP telomere interactors from WT cell lysates form interaction clusters. Biogrid protein interactions are represented as a Heatmap. Yellow is used for presence, and black is used for absence of an annotated interaction. Clustering is performed using the complete data based on binary distance. Npl3 interactors are highlighted in red.

B Annotated RNA interaction motifs for Npl3.

C Npl3-TAP is functional. npl3 cells are temperature sensitive at 37°C. This sensitivity is not observed in WT or Npl3-TAP cells. Serial dilutions of indicated strains were assayed on YPD. Cells were plated at indicated temperatures and grown for 48 h.

D Npl3-TAP association to telomeres does not change between telomerase-positive and tlc1 cells when cells are propagated after dissection for 60 population doublings. Cross-linked samples from indicated strains were used in a TAP-ChIP. Enrichment at telomeres was determined by quantitative PCR on indicated telomeres. Data represent mean % input ± SEM relative to cells arrested in alpha factor n = 3 (unpaired t-test, two-tailed *P < 0.05, **P < 0.01). PD60 refers to 60 population doublings in the absence of TLC1 (telomerase RNA subunit).

E Npl3-TAP protein levels do not change in tlc1 cells. Protein levels are determined using PAP (to detect the TAP tag) and anti-actin antibodies for loading comparison.

Data information: PAP, peroxidase anti-peroxidase; PD, population doublings; TAP, tandem affinity purification tag.

Figure EV2. RNA-dependent telomere-associated proteins in yeast.

A Heatmap for enriched proteins at the telomere bait in WT and tlc1 cells. Color code indicates measured intensities in the telomere pull-down. Heatmap was constructed using the "pheatmap" package in R, where clustering is performed using complete data based on the Euclidean distance. Gray indicates not enriched.

B Heatmap for enriched proteins at the telomere bait tlc1 cells and tlc1 cells treated with RNAse A and RNase H. Color code indicates measured intensities in the telomere pull-down. Heatmap was constructed using the "pheatmap" package in R, where clustering is performed using complete data based on the Euclidean distance. Gray indicates not enriched.

C RNA-dependent telomere interactors in tlc1 cell lysates form protein interaction clusters. Proteins identified exclusively in tlc1 non-treated pull-downs when compared to tlc1 RNase A- and RNase H-treated extracts were used for the analysis (i.e., the RNase sensitive interactors). Biogrid protein interactions are represented as a Heatmap. Proteins with less than five physical interactions were filtered out. Yellow is used for presence and black for absence of interaction. Clustering is performed using the complete data based on binary distance. Npl3 interactors are highlighted in red.

 © 2020 The Authors

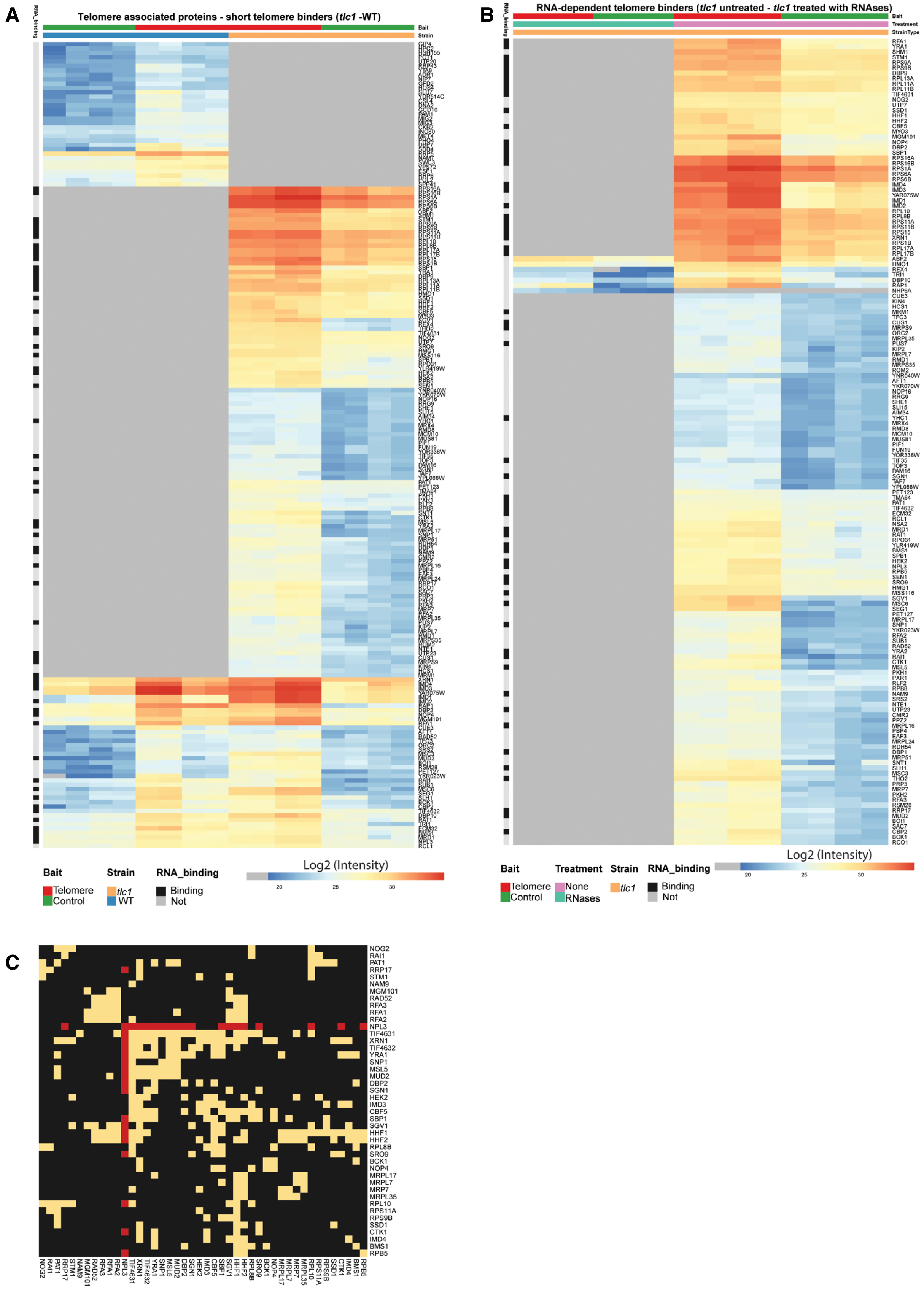
A
Telomere associated proteins - short telomere binders (tlc1 -WT)
RNA_binding
Bait
Strain
Bait
Telomere
Control
Strain
tlc1
WT
RNA_binding
Binding
Not
Log2 (Intensity)
20
25
30
B
RNA-dependent telomere binders (tlc1 untreated - tlc1 treated with RNAses)
Bait
Treatment
StrainType
Bait
Telomere
Control
Treatment
None
RNases
Strain
tlc1
RNA_binding
Binding
Not
Log2 (Intensity)
20
25
30
C

Figure EV2.

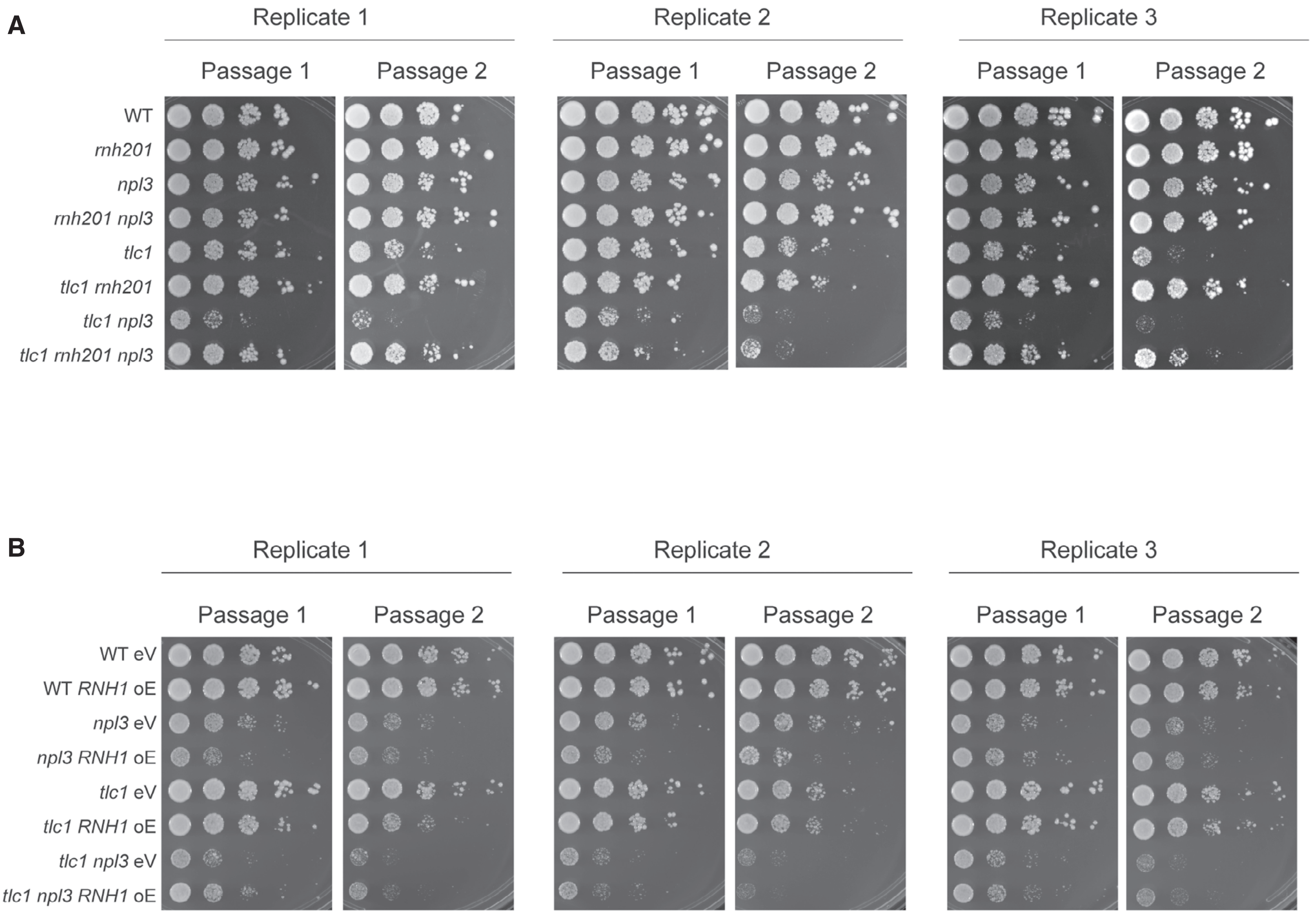

Figure EV3. Npl3 regulates senescence rate.

A Serial dilutions of indicated strains were assayed on YPD media. Cells were plated after 30–50 generations propagated on YPD agar media. Two consecutive passages from 3 different biological replicates are shown. Plates were imaged after 72 h growth at 30°C.

B Serial dilutions of indicated strains were assayed on SC-His media. Cells were plated after 30–50 generations propagated on SC-His agar media. Two consecutive passages from three different biological replicates are shown. Plates were imaged after 72 h growth at 30°C.

© 2020 The Authors
