# Supplementary material for: Npl3 stabilizes R‐loops at telomeres to prevent accelerated replicative senescence
Source: EMBO Rep. 2020 Feb 6;21(3):e49087. doi: 10.15252/embr.201949087 (PMC7054685; doi:10.15252/embr.201949087)
Supplement: Supplementary file 2 — Table EV1 [file EMBR-21-e49087-s002.docx]

## **Table EV1: Yeast strains used in this study.**

| **Strain n.** | **Name** | **Genotype** | **Source** |
| --- | --- | --- | --- |
| yBL7 | wildtype S288C | MATa *his3Δ1 leu2Δ0 ura3Δ0 met15Δ0* | Euroscarf |
| yMD1245 | *TLC1*/*tlc1* | S288C Mat a/Mat α Tlc1/tlc1::his | [19] |
| yLP10 | *tlc1*  *NPL3-TAP* | S288C Mat a/Mat α *TLC1/tlc1::Nat*  *NPL3/NPL3-TAP-HIS3MX6* | This study |
| yLP16 | *NPL3-TAP* | S288C Mat a *NPL3-TAP-HIS3MX6* | Dharmacon |
| yLP130 | *tlc1*  *npl3* +pBL335 (eV) | S288C Mat a/Mat α *TLC1/tlc1::Nat*  *NPL3/npl3*  +pBL335 | This study |
| yLP131 | *tlc1*  *npl3* +pBL336 (*RNH1* oE) | S288C Mat a/Mat α *TLC1/tlc1::Nat*  *NPL3/npl3*  +pBL336 (*RNH1*  oE) | This study |
| yLP193 | *NPL3-TAP*  *RAT1-AID*  *bar1*  *afb2* | S288C Mat α *NPL3-TAP-HIS3MX6*  *RAT1-AID-HYG*; *bar1::KAN; afb2::LEU2* | This study |
| yLP202 | *RAT1-AID*  *bar1*  *afb2* | S288C Mat α  *RAT1-AID-HYG*; *bar1::KAN; afb2::LEU2* | This study |
| yLP303 | *NPL3-TAP* +eV | S288C Mat a *NPL3-TAP-HIS3MX6*  +pBL211(eV) | This study |
| yLP305 | *NPL3-TAP* + *RNH1* oE | S288C Mat a *NPL3-TAP-HIS3MX6*  +pBL352 (*RNH1* oE) | This study |
| yLP307 | wildtype S288C +eV | S288C Mat a +pBL211(eV) | This study |
| yLP315 | *tlc1*  *npl3*  *rad52* | S288C Mat a/Mat α *TLC1/tlc1::Nat*  *RAD52/rad52::HIS3; NPL3/npl3::KAN* | This study |
| yLP309 | wildtype S288C +*RNH1*  oE | S288C Mat a +pBL352(*RNH1* oE) | This study |
| yLP380 | *rnh201*  *tlc1*  *npl3* | S288C Mat a/Mat α *TLC1/tlc1::Nat*  *RNH201/rnh201::HYG; NPL3/npl3::KAN* | This study |
| yLP400 | *npl3*  *tlc1* +*NPL3*  oE | S288C Mat a/Mat α *TLC1/tlc1::Nat*  *NPL3/npl3::KAN* +pBL565 (pRD54_*NPL3* oE) | This study |
| yLP550 | wildtype S288C +eV pRD54 | S288C Mat a +pBL19(pRD54_eV) | This study |
| yLP551 | wildtype S288C +*NPL3* oE | S288C Mat a +pBL565 (pRD54_*NPL3* oE) | This study |
| yLP679 | *sen1-1* +eV pRD54 | S288C Mat a *sen1-1::KAN* +pBL19(pRD54_eV) | This study |
| yLP681 | *sen1-1* +*NPL3* oE | S288C Mat a *sen1-1::KAN* +pBL565 (pRD54_*NPL3* oE) | This study |
