# Supplementary material for: Npl3 stabilizes R‐loops at telomeres to prevent accelerated replicative senescence
Source: EMBO Rep. 2020 Feb 6;21(3):e49087. doi: 10.15252/embr.201949087 (PMC7054685; doi:10.15252/embr.201949087)
Supplement: Supplementary file 3 — Table EV2 [file EMBR-21-e49087-s003.docx]

## **Table EV2: Primers used in this study.**

| **Oligo n.** | **Use** | **Sequence (5’-3’)** |
| --- | --- | --- |
| oBL295 | qPCR -1L telomere | CGGTGGGTGAGTGGTAGTAAGTAGA |
| oBL296 | qPCR -1L telomere | ACCCTGTCCCATTCAACCATAC |
| oBL292 | qPCR- actin | CCCAGGTATTGCCGAAAGAATGC |
| oBL293 | RT and qPCR-actin | TTTGTTGGAAGGTAGTCAAAGAAGCC |
| oLK57 | qPCR-15L | GGGTAACGAGTGGGGAGGTAA |
| oLK58 | qPCR-15L | CAACACTACCCTAATCTAACCCTGT |
| oLK49 | qPCR- 6Y’ | GGCTTGGAGGAGACGTACATG |
| oLK50 | qPCR- 6Y’ | CTCGCTGTCACTCCTTACCCG |
| oBL207 | TERRA RT | CACCACACCCACACACCACACCCACA |
| oBL29 | Confirm *NPL3* KO | CTGCAGCGAGGAGCCGTAAT |
| oLP8 | Confirm *NPL3* KO | GGCTTATTGATTACAATTGCTTGTT |
| oLP115 | Telomere bait | GTGGGTGTGTGGTGTGGGTGTGTGGGTGTGTGTGGTGTGGGTGTGTGTGGGTGTGTGTGTGGGTGTGGGTGTGGTGT |
| oLP116 | Telomere bait | ACACACCACACCCACACCCACACACACACCCACACACACCCACACCACACACACCCACACACCCACACCACACACCC |
| oLP117 | Control bait | GTGAGTGTGAGTGTGAGTGTGAGTGTGAGTGTGAGTGTGAGTGTGAGTGTGAGTGTGAGT |
| oLP118 | Control bait | ACACTCACACTCACACTCACACTCACACTCACACTCACACTCACACTCACACTCACACTC |
| oLP134 | Cloning *NPL3* into pRD54 | AGGGCTGCAGGAATTCTCTGAAGCTCAAGAAACTCACGT |
| oLP135 | Cloning *NPL3* into pRD54 | GCTTGATATCGAATTCAAACTTATTTTTGTTAATTTTGCAGCACAT |
| oAM47 | 18SrDNA qPCR | TCC AAT TGT TCC TCG TTA AG |
| oAM48 | 18SrDNA qPCR | ATT CAG GGA GGT AGT GAC AA |
| oMG59 | SCR1 RT (7S RNA) | GGCAGGAGGCGTGAGGAATC |
| oMG60 | SCR1 RT and qPCR (7S RNA) | CCTAACAGCGGTGAAGGTGGAG |
