# Supplementary material for: Npl3 stabilizes R‐loops at telomeres to prevent accelerated replicative senescence
Source: EMBO Rep. 2020 Feb 6;21(3):e49087. doi: 10.15252/embr.201949087 (PMC7054685; doi:10.15252/embr.201949087)
Supplement: Supplementary file 4 — Table EV3 [file EMBR-21-e49087-s004.docx]

## **Table EV3: Plasmids used in this study.**

| **Recombinant DNA** | | |
| --- | --- | --- |
| Plasmid: pRS425- GAL, 2u, *LEU2* | [15] | pBL211 |
| Plasmid: pRS425-  *RNH1-HA,* GAL, 2u, *LEU2*, | [15] | pBL352 |
| Plasmid: pRD54 GAL-HA, CEN, *URA3* | Matthias Peter | pBL19 |
| Plasmid: pRD54 *NPL3-HA*, GAL, CEN, *URA3* | This study | pBL565 |
| Plasmid: pT316 GPD, CEN, *HIS3* | Teixiera lab | pBL335 |
| Plasmid: pT316 RNH1 GPD, CEN, HIS3 | Teixiera lab | pBL336 |
| Plasmid: pSP100, contains probe for Southern Blot | Longhese lab | pBL423 |
