# Supplementary material for: Npl3 stabilizes R‐loops at telomeres to prevent accelerated replicative senescence
Source: EMBO Rep. 2020 Feb 6;21(3):e49087. doi: 10.15252/embr.201949087 (PMC7054685; doi:10.15252/embr.201949087)
Supplement: Supplementary file 5 — Table EV4 [file EMBR-21-e49087-s005.docx]

## **Table EV4: Antibodies used in this study.**

| Reagent | Supplier | Identifier |
| --- | --- | --- |
| **Antibodies** | | |
| Mouse anti-FLAG M2 | Sigma-Aldrich | Cat. Number F3165 |
|  |  | RRID:AB_259529 |
| Rabbit PAP (Peroxidase Anti-peroxidase soluble complex) | Sigma-Aldrich | Cat. number |
|  |  | RRID:AB_1079562 |
| Mouse monoclonal anti-Actin Clone C4 | Millipore | Cat. number MAB1501R |
|  |  | RRID:AB_2223041 |
| Mouse monoclonal anti-HA.11 Clone 16B12 | Covance | Cat. number MMS-101P |
|  |  | RRID:AB_2314672 |
| Mouse monoclonal anti-DNA-RNA Hybrid (S9.6) | Kerafast | Cat. number ENH001; RRID:AB_2687463 |
| Mouse monoclonal anti-ds DNA (35I9 DNA) | Abcam | Cat. number ab27156; RRID:AB_470907 |
| Goat Immun-Star anti-mouse (GAM)-HRP conjugate | Bio-Rad | Cat. number 170-5047; RRID: AB_11125753 |
